# The Process-Outcome Mindfulness Effects in Trainees (PrOMET) study: protocol of a pragmatic randomized controlled trial

**DOI:** 10.1186/s40359-015-0082-3

**Published:** 2015-07-17

**Authors:** Johannes Mander, Paula Kröger, Thomas Heidenreich, Christoph Flückiger, Wolfgang Lutz, Hinrich Bents, Sven Barnow

**Affiliations:** University of Heidelberg, Center for Psychological Psychotherapy, Bergheimer Str. 58a, 69115 Heidelberg, Germany; Department of Social Work, Health and Nursing, University of Applied Sciences Esslingen, Esslingen, Germany; Department of Clinical Psychology and Psychotherapy, University of Bern, Bern, Switzerland; Department of Clinical Psychology and Psychotherapy, University of Trier, Trier, Germany; Department of Clinical Psychology and Psychotherapy, University of Heidelberg, Heidelberg, Germany

**Keywords:** Mindfulness, Therapeutic alliance, Psychotherapy, Randomized controlled trial, Multilevel models

## Abstract

**Background:**

Mindfulness has its origins in an Eastern Buddhist tradition that is over 2500 years old and can be defined as a specific form of attention that is non-judgmental, purposeful, and focused on the present moment. It has been well established in cognitive-behavior therapy in the last decades, while it has been investigated in manualized group settings such as mindfulness-based stress reduction and mindfulness-based cognitive therapy. However, there is scarce research evidence on the effects of mindfulness as a treatment element in individual therapy. Consequently, the demand to investigate mindfulness under effectiveness conditions in trainee therapists has been highlighted.

**Methods/Design:**

To fill in this research gap, we designed the PrOMET Study. In our study, we will investigate the effects of brief, audiotape-presented, session-introducing interventions with mindfulness elements conducted by trainee therapists and their patients at the beginning of individual therapy sessions in a prospective, randomized, controlled design under naturalistic conditions with a total of 30 trainee therapists and 150 patients with depression and anxiety disorders in a large outpatient training center. We hypothesize that the primary outcomes of the session-introducing intervention with mindfulness elements will be positive effects on therapeutic alliance (Working Alliance Inventory) and general clinical symptomatology (Brief Symptom Checklist) in contrast to the session-introducing progressive muscle relaxation and treatment-as-usual control conditions. Treatment duration is 25 therapy sessions. Therapeutic alliance will be assessed on a session-to-session basis. Clinical symptomatology will be assessed at baseline, session 5, 15 and 25. We will conduct multilevel modeling to address the nested data structure. The secondary outcome measures include depression, anxiety, interpersonal functioning, mindful awareness, and mindfulness during the sessions.

**Discussion:**

The study results could provide important practical implications because they could inform ideas on how to improve the clinical training of psychotherapists that could be implemented very easily; this is because there is no need for complex infrastructures or additional time concerning these brief session-introducing interventions with mindfulness elements that are directly implemented in the treatment sessions.

**Trial registration:**

From ClinicalTrials.gov, Identifier: NCT02270073 (registered October 6, 2014)

## Background

Mindfulness has its origins in an Eastern Buddhist tradition that is over 2500 years old and can be conceptualized as a specific form of attention that is non-judgmental, purposeful, and focused on the present moment (Cigolla and Brown 2011; Heidenreich and Michalak 2003). Currently, it is well established in cognitive-behavior therapy (CBT), with mindfulness-based stress reduction (MBSR; (Kabat-Zinn 1990; Grossman et al. 2004)) as its most prominent application. Usually, mindfulness interventions have been investigated in a structured, manualized group setting (Bohlmeijer et al. 2010). However, an increasing demand to further investigate the effects of mindfulness practiced together by patients and therapists in individual therapy under naturalistic conditions has been highlighted to address external validity issues (e.g. (Ryan et al. 2012; Bruce et al. 2010)).

### Therapist mindfulness practice and therapeutic alliance

Concerning the therapist’s perspective, it has been highlighted that qualities such as empathy, understanding, warmth, genuineness, and acceptance have been found to be important for developing a positive therapeutic alliance and for predicting therapeutic outcome (Lambert and Barley 2002; Orlinsky et al. 2004). Furthermore, they have been found to be positively related to mindfulness practice (Cigolla and Brown 2011; Horst et al. 2013). Consequently, a two-stage model concerning the association between mindfulness and the activation of a therapeutic alliance has been hypothesized: Mindfulness practice improves self-acceptance and self-compassion as preconditions for the further development of empathy and acceptance towards patients (Bruce et al. 2010; Kristeller and Johnson 2005; Siegel 2007). Consequently, the strength of the therapeutic alliance might be enhanced (Horst et al. 2013).

### Patient mindfulness practice and explanation of clinical improvements

Concerning the patient’s perspective, it has been hypothesized that mindfulness practice leads to a better sense of self-acceptance, better emotion regulation, increased body awareness, and an improvement of interpersonal relationships (Bruce et al. 2010; Hölzel et al. 2011). This in turn may explain the associations between patients’ mindfulness practice and improvements in therapeutic alliance, general wellbeing, emotional clarity, self-esteem, and life satisfaction that have been identified by several studies (Brown and Ryan 2003; Shapiro et al. 2008; Baer et al. 2004; Dunn et al. 2013).

### Mindfulness in effectiveness research in trainee therapists

In the context of investigating the mutual mindfulness experiences of patients and therapists, it has been specifically highlighted that brief, in-session mindfulness interventions should be investigated in trainee therapists (Dunn et al. 2013; Grepmair et al. 2007), as this would elucidate opportunities to improve clinical training (Ryan et al. 2012).

Nevertheless, only a few studies have been conducted addressing these limitations: In an inpatient study, the patients of therapists in training who received a 2-month Zen-meditation workshop program showed a stronger symptom reduction than a control group did (Grepmair et al. 2007). Another study demonstrated that trainee therapists’ dispositional mindfulness did not directly predict therapeutic outcome but was associated with improvements in the interpersonal functioning of the patient and the therapeutic alliance (Ryan et al. 2012). Additionally, an analysis of interview transcripts in a qualitative study demonstrated that the shared experience of mindfulness of both patient and trainee therapist led to improvements in therapeutic alliance and was helpful with transitions from everyday activity to therapy sessions (Horst et al. 2013). Furthermore, a study in which trainee therapists conducted a 5-min mindfulness-centering exercise before therapy sessions resulted in therapists perceiving themselves as being more present and in patients perceiving therapeutic alliances as being stronger and therapy as being more effective compared to a control group (Dunn et al. 2013). This cross-sectionally designed study highlights that it seems to be auspicious to implement brief mindfulness interventions in a longitudinally designed, randomized controlled trial of therapists in training. However, there is also a study that demonstrated a negative association of trainee therapists’ self-reports of dispositional levels of mindfulness and therapeutic outcomes (Stanley et al. 2006).

### Mindfulness studies with active control groups

In spite of this preliminary evidence of positive effects of mindfulness on therapeutic alliance, little is known about the specific mechanisms of mindfulness in psychotherapy (Coffey et al. 2010). Furthermore, only a few studies have compared mindfulness interventions with an active control group, and most of the investigations that did were conducted with non-clinical samples. Most studies applied progressive muscle relaxation (PMR) as an active control group and demonstrated the beneficial effects of mindfulness (e.g. (Feldman et al. 2010; Semple 2010)). Hence, randomized controlled trials with a focus on associations of mindfulness with therapeutic alliance from both the patient and therapist perspectives are needed (Ryan et al. 2012; Bruce et al. 2010; Horst et al. 2013). This research should be conducted under naturalistic conditions, because effectiveness studies are generally demanded by leading psychotherapy researchers to address issues of external validity (Orlinsky et al. 2004; Lambert 2013; Norcross and Lambert 2011; Lutz 2003; Flückiger et al. 2012).

### Objectives

The main purpose of this study is to identify whether exercises with mindfulness elements carried out at the beginning of individual therapy sessions help to improve the therapeutic process under effectiveness conditions. Consequently, we designed a study to analyze the effects of a 5-min session-introducing intervention with mindfulness elements (SIIME) conducted together by trainee therapists and their patients in a randomized, controlled, longitudinal design under naturalistic conditions with PMR as the active control group.

More specifically, we will examine the effects of SIIME practiced by both outpatients and CBT trainee therapists at the beginning of the 25 therapy sessions (brief-term CBT of the German healthcare system) on (a) therapeutic alliance measured on a session-to-session basis and (b) on clinical outcomes assessed every 10 sessions. Thus, before the start of therapeutic treatment, patients are randomized into a treatment-as-usual + mindfulness intervention group (TAU + M) practicing SIIME, a TAU + PMR-control group practicing a session-introducing short form of PMR, or a TAU control group without standardized session-introducing intervention. Before the start of the intervention study, all therapists will participate in a 6-week workshop-based mindfulness and PMR program. We will investigate the following hypotheses:

1. We hypothesize that both patients and therapists of the TAU + M will experience higher levels of therapeutic alliance compared to the TAU + PMR and TAU.
2. We hypothesize that in the TAU + M, there will be stronger reductions in clinical symptomatology of patients compared to the TAU + PMR and TAU.

The results of the planned study are important in three ways. First, they will complement earlier preliminary evidence on the effects of mindfulness on therapeutic alliance and clinical improvements by addressing the above-mentioned research gaps in effectiveness research. Second, they will more specifically elucidate the potential beneficial effects of brief SIIME as a tool to introduce therapy sessions. Third, they could provide important practical implications: The results could deliver ideas about how to improve the training of psychotherapists in outpatient training centers. If they are replicated in other studies, positive results could lead to important developments in modifications of psychotherapy training that could be implemented very easily, as there is no need for complex infrastructures or additional time concerning these SIIME that are directly implemented in the treatment sessions.

## Method

### Standard procedures at the Center for Psychological Psychotherapy

The Center for Psychological Psychotherapy (CPP) is a large university outpatient training center for CBT at the University of Heidelberg. Approximately 1000 patients per year with different types of psychiatric disorders (about two-thirds suffering from anxiety and depression) are treated there by approximately 100 trainee therapists. The CPP has 14 rooms for providing psychotherapy. All rooms offer the opportunity to videotape sessions and to listen to audiotapes.

The training of therapists at the CPP follows the standard procedures of CBT formation in Germany. Specifically, all trainees receive a minimum of 600 h of theory, 150 h of supervision, and 120 h of self-experience, and they perform 600 h of outpatient therapy. Furthermore, before beginning with individual outpatient therapy, they perform 18 months of internships in psychiatric and psychosomatic hospitals. All outpatient therapy sessions are supervised by accredited experts. Outpatient sessions begin after a six-session diagnostic stage and are not based primarily on specific CBT treatment manuals but rather on individual case formulations as developed by trainees in collaboration with their supervisors to guarantee the optimal adaptation of therapeutic interventions for the individual needs of patients. Outcome assessments at the baseline, at every tenth session during treatment, at the end of therapy, and at a 12-month follow-up are standard procedure at the CPP.

### Sample and ethics

A total of 30 trainee therapists and 150 patients will be recruited at the CPP. Enlisting as a trainee in the clinic is preceded by a rigorous assessment that evaluates the potential trainee’s personal suitability for becoming a CBT therapist.

The general inclusion criterion for patients is a primary depressive- or anxiety-disorder diagnosis in the Structured Clinical Interview according to the DSM-5 criteria (Falkai et al. 2014). We chose these two disorder groups because from patients being treated at German university therapy-training centers; approximately 40 % suffer from a primary major depression, and approximately 30 % suffer from a primary anxiety disorder (e.g. (Nelson and Hiller 2013)). Hence, our results will be of importance to a majority of outpatient diagnostic groups.

The general exclusion criteria for patients will be as follows: (1) an age below 18 or above 65 years, (2) insufficient German language skills, (3) those suffering from a psychotic disorder, (4) current suicidal risk. Comorbidities with disorders not on the exclusion list are generally not considered as limitations to entering the study, as long as depression and anxiety disorders are of primary concern.

Power analyses with G*Power (Faul et al. 2007) for the detection of small effects (Cohen’s *f* = 0.12) for the interaction between time (pre, mid-5, mid-15, post) and treatment condition (TAU-M versus TAU-PMR versus TAU) (ANOVA repeated measures, within-between-interaction, α = 0.05, power = 0.80, number of groups = 3, number of measurements = 4, pre-post correlation *r* = 0.05, nonsphericity correction = 1) along with practical clinical considerations (Flückiger 2014) resulted in a sample size of 123 patients. Taking potential dropouts into account, a total sample of 150 patients will be assigned to one of the three groups by a stratified randomization process. Patients will be stratified into one of two categories: one group with the main diagnosis of major depression and the other group with the main diagnosis of an anxiety disorder.

Voluntary participation and written, informed consent are necessary conditions for participation in the study. The local ethics committee (Ethikkommission der Fakultät für Verhaltens- und Empirische Kulturwissenschaften der Universität Heidelberg) approved the study protocol in accordance with the Helsinki Declaration.

### Mindfulness workshop training

To prepare the 30 trainee therapists for the SIIME outlined in the next section, we will first offer two workshops separated by a 6-week home-practice interval to all trainee therapists participating in the study. The workshops will be offered twice every year when a new group of trainee therapists starts their outpatient therapies. The first workshop will provide a theoretical background in mindfulness, including descriptions of its roots in Theravada Buddhism, its first practical implications in MBSR, and the integration of mindfulness in the other third-wave approaches of CBT. Furthermore, formal mindfulness practices (specific techniques such as breathing meditation and body scan) and informal mindfulness practices (introducing mindfulness in everyday life by mindfully carrying out activities like showering, eating, and walking) will be described and conducted. In the 6-week interval between workshops one and two, participants will practice formal and informal mindfulness activities at home. The second workshop will specifically address the mindfulness experiences of participants, and suggestions for further improvements will be the focus. Additionally, to reduce the potential allegiance effects in relation to mindfulness interventions, all participating therapists will receive two regular workshops on relaxation techniques that specifically focus on PMR as part of their regular therapist-training program.

### Experimental session

The experiment will be conducted at the CPP university training center. Before conducting the first intervention, a standardized text concerning reasons for the relevant condition (SIIME, PMR or TAU) will be presented. Additionally, all components of the relevant intervention will be explained and practiced.

During the 5-min mindfulness experimental task, both patient and therapist of the TAU + M sit at a distance of about 1 m from the audio recorder. After the initial greeting ritual, both patient and therapist together perform the SIIME for the first 5 min of the therapy session. While performing the exercise, patient and therapist sit upright in their chairs in a comfortable position with their feet flat on the floor, their arms and legs uncrossed, and their hands resting in their laps. The text of the intervention is standardized and spoken by Dr. Thomas Heidenreich (TH), an internationally renowned expert on mindfulness research. During the exercise, both patient and therapist are instructed to mindfully observe their breathing and body sensations. After completion of the SIIME, the regular therapy session begins. Following the treatment session, both patient and therapist complete the session questionnaire described in the following section, which requires about two minutes.

The TAU + PMR also receives a 5-min audiotaped exercise under basically the same conditions; more specifically, they will receive a short version of PMR that is also spoken by TH. On the one hand and as mentioned above, PMR is a broadly accepted and easy-to-implement relaxation exercise that is applied most often as a control intervention when investigating mindfulness interventions. On the other hand, it does not include the hypothesized specific effective ingredients of the SIIME (mindful observation and acceptance of physiological and psychological conditions). The wording of the control intervention is as similar to the experimental intervention as possible.

In the first therapy sessions, a brief Inquiry (brief exploration about experiences during the exercises) will be conducted after the SIIME and session-introducing PMR intervention. As required, the Inquiry can be conducted in later therapy sessions, too. The TAU-CG will receive standard individual therapy sessions as usual at the CPP.

### Development and feasibility of the intervention

The SIIME was developed by an iterative process in multiple steps: First, TH and Johannes Mander (JM) phrased a preliminary version based on the breathing-space exercise by Michalak, Heidenreich, and William (Michalak et al. 2012). Then, improvements were performed based on the mindfulness centering exercise by Eifert and Forsyth (2005) that was used in the above-mentioned study by Dunn et al. (2013). Furthermore, 10 therapists and 10 patients conducted this preliminary exercise in one therapy session and delivered feedback. After improving the exercise according to patient and therapist feedback, five mindfulness experts and two experts in psychotherapy process research reviewed the exercise and offered feedback for improvement. Based on these feedback processes, the final version of the SIIME was developed. The PMR control condition was developed based on the same procedure.

The complete wording of both interventions is listed in the Appendix A and Appendix B.

The feasibility of the interventions was then tested in a pre-study with 12 therapists and 12 patients. Both patients and therapists conducted the exercises at the beginning of one therapy session. Additionally, they completed questions concerning the feasibility of the interventions. They reported on a rating scale from 0 (does not apply) to 4 (fully applies) that both interventions could be integrated without problems in the everyday therapeutic process (M = 3.38; SD = 0.56), that the instructions were understandable (M = 3.51; SD = 0.51), and that the exercises generally had a positive impact on the therapy session (M = 2.43; SD = 0.96).

### Blinding, allegiance, and randomization

The focus of the current study is on the external validity and generalizability of the results to routine clinical practice. Consequently, according to the extension of the CONSORT statement concerning the criteria of a pragmatic randomized, controlled trial (Zwarenstein et al. 2008), no blinding concerning treatment conditions will be implemented in the current study. All participants will be informed about the general aims and procedures of the study. However, they will be blind concerning the specific hypotheses.

In our study, we will apply a crossed-therapist design in which a given therapist delivers the experimental and control conditions (Falkenstrom et al. 2013). This design includes specific limitations (e.g., the increased problem of allegiance effects (Falkenstrom et al. 2013)), but will be necessary to conduct the study in routine care. To address the potential problems of this design, we will control for allegiance effects with a specific instrument. Furthermore, all three treatment conditions will be introduced in a neutral way, and preparation in PMR will be as intensive as mindfulness preparation. Additionally, the crossed-therapist design allows researchers to address the issue of potential therapist effects (Baldwin and Imel 2013). More specifically, as efficacy varies across therapists, a crossed-therapist design allows researchers to control for these effects as the same therapists perform all of the different treatment conditions.

**Fig. 1:**
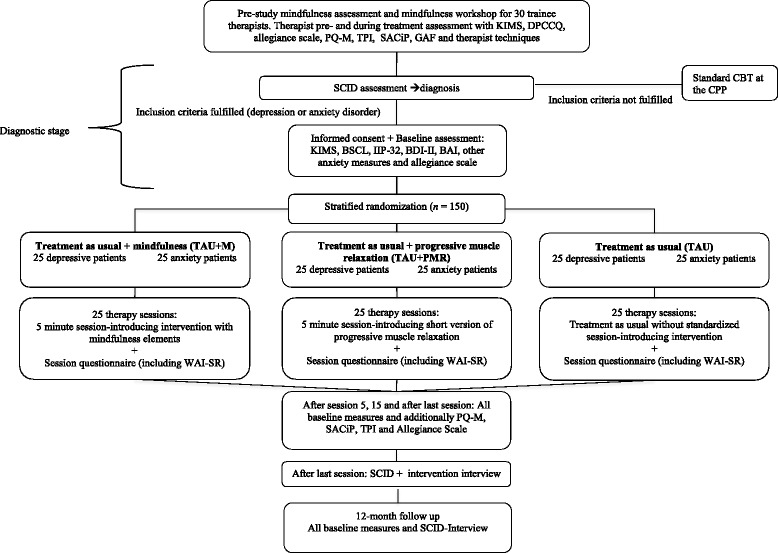
Study design and measurement time points (CONSORT chart). BAI = Beck Anxiety Inventory; BDI-II = Beck Depression Inventory II; BSCL = Brief Symptom Checklist; DPCCQ = Development of Psychotherapists Common Core Questionnaire; GAF = Global Assessment of Functioning; IIP-32 = Inventory of Interpersonal Problems; KIMS = Kentucky Inventory of Mindfulness Skills; PQ-M = Practice Quality-Mindfulness; SACiP = Scale for the Multiperspective Assessment of General Change Mechanisms in Psychotherapy; SCID = Structured Clinical Interview for DSM; TPI = Therapeutic Presence Inventory; WAI-SR = Working Alliance Inventory-Short Revised; CPP = Center for Psychological Psychotherapy

Treatment allocation will be performed by a blocked and stratified randomization process with a computerized random-number generator (www.random.org). Patients will be stratified according to their main diagnosis (depression or anxiety disorder) and then randomized by a balanced blocking procedure into the three treatment arms. Randomization will be conducted by an independent research assistant, while the other researchers have no access to the randomization list and process. According to the CONSORT statement (Altman et al. 2001), the people involved in the generation and implementation of the random treatment sequences will be completely separated. A CONSORT flow chart of the study design is depicted in Fig. 1.

### Dependent variables

For an overview of the assessment measures, see Table 1.

**Table 1:** Application plan of measures

Measures			Measurement waves			
	Pre	Session by session	Mid-5	Mid-15	Post	Follow Up
**Clinical assessment- therapists**						
Global Assessment of Functioning (GAF)	+		+	+	+	+
Development of Psychotherapists Common Core Questionnaire (DPCCQ)	+				+	+
Scale for the Multiperspective Assessment of General Change Mechanisms in Psychotherapy-Therapist (SACiP-T)			+	+	+	
Therapist techniques			+	+	+	
Allegiance scale	+		+	+	+	+
**Clinical assessment- patients**						
Structured Clinical Interview for DSM (SCID)	+				+	+
Brief Symptom Checklist (BSCL)	+		+	+	+	+
Inventory of Interpersonal Problems (IIP)	+		+	+	+	+
Beck Depression Inventory (BDI-II)	+		+	+	+	+
Beck Anxiety Inventory (BAI)	+		+	+	+	+
Scale for the Multiperspective Assessment of General Change Mechanisms in Psychotherapy-Patient (SACiP-P)			+	+	+	
Therapist techniques and study interventions (Interview by research team)					+	
Allegiance scale	+		+	+	+	+
**Mindfulness assessment-therapists**						
Kentucky Inventory of Mindfulness Skills-Therapist (KIMS-T)	+		+	+	+	+
Therapist Presence Inventory-Therapist (TPI-T)			+	+	+	
Practice Quality-Mindfulness-Therapist (PQ-M-T)			+	+	+	
**Mindfulness assessment-patients**						
Kentucky Inventory of Mindfulness Skills-Patient (KIMS-P)	+		+	+	+	+
Therapist Presence Inventory-Patient (TPI-P)			+	+	+	
Practice Quality-Mindfulness-Patient (PQ-M-P)			+	+	+	
**Session questionnaire-therapists**						
Working Alliance Inventory-Short Revised-Therapist (WAI-SR-T)		1–25				
Therapist Presence Inventory-Therapist-Short (TPI-T-S)		1–25				
Practice Quality-Mindfulness-Therapist-Short (PQ-M-T-S)		1–25				
**Session questionnaire-patients**						
Working Alliance Inventory- Short Revised- Patient (WAI-SR-P)		1–25				
Therapist Presence Inventory-Patient-Short (TPI-P-S)		1–25				
Practice Quality-Mindfulness-Patient-Short (PQ-M-P-S)		1–25				

### Primary outcome measures

- Primary outcome measure 1: To assess the quality of the therapeutic alliance, we will apply the Working Alliance Inventory- Short Revised (WAI-SR; (Hatcher and Gillaspy 2006)). The WAI-SR is a 12-item self-report instrument with patient and therapist perspectives rated on a seven-point scale. It includes three subscales: bond, goals, and tasks. The bond subscale reflects the emotional relationship between patient and therapist. The goals and tasks subscales refer to the agreement of patients and therapists concerning the treatment goals and tasks. The WAI-SR is considered the gold standard for alliance assessment, has excellent psychometric properties, and is outcome predictive, as has been demonstrated in meta-analyses (e.g. (Horvath et al. 2011)).
- Primary outcome measure 2: To assess general symptom severity, the Global Severity Index (GSI) of the Brief Symptom Checklist (BSCL; (Derogatis and Melisaratos 1983; Franke 2000)) will be applied. The BSCL is the short version of the Symptom-Checklist-90-Revised (SCL-90-R; (Derogatis and Lazarus 1994)) and consists of 53 items forming nine subscales as follows: somatization, obsessive-compulsive, interpersonal sensitivity, depression, anxiety, hostility, phobic anxiety, paranoid ideation, and psychoticism, which are rated on a five-step scale. It showed excellent internal consistencies, with 0.71 ≤ α ≤ 0.85; good retest reliabilities, with 0.68 ≤ *r* ≤ 0.91; high correlations to the original SCL-90-R, with 0.92 ≤ *r* ≤ 0.99; and good construct validity, with scale-outcome correlations between 0.30 ≤ *r* ≤ 0.72.

### Secondary outcome measures

- To assess general depressive symptoms of the patients, we will apply the Beck Depression Inventory-II (BDI, (Hautzinger et al. 1994; Beck et al. 1996)), a screening instrument for depression derived from the criteria of the DSM-IV (American Psychiatric Association 2000) that consists of 21 items on a four-step scale. It revealed an internal consistency of *α* = 0.88, a split-half reliability of *r* = 0.72, a retest reliability of *r* = 0.75, and convergent validities of 0.71 ≤ *r* ≤ 0.89.
- To assess general anxiety symptoms of the patients, we will apply the Beck Anxiety Inventory (BAI; (Beck et al. 1988; Margraf and Ehlers 2007)), which consists of 21 items. It revealed an internal consistency of *α* = 0.90, a split-half reliability of *r* = 0.70, a retest reliability of *r* = 0.75, and convergent validities of 0.50 ≤ *r* ≤ 0.61.
- To assess interpersonal functioning, we will apply the short version of the Inventory of Interpersonal Problems (IIP), which is a 32-item instrument with a circumplex structure (Horowitz et al. 1988). It consists of eight factors that are rated on a five-step scale: domineering, intrusive, overly nurturant, exploitable, nonassertive, socially avoidant, cold, and vindictive. It has excellent psychometric properties, with 0.75 ≤ α ≤ 0.94. The criterion validity of the measure has been demonstrated via correlations to the SCL-90-R with 0.07 ≤ *r* ≤ 0.75.
- To assess therapists’ views on the psychological, social, and occupational functioning of the patients, we will apply the Global Assessment of Functioning (GAF). This 100-point scale is divided into sections, each with ten points. The ten-point intervals have anchor points (verbal instructions) describing symptoms and functioning, while the 1–10 interval describes the most severely ill and the 91–100 interval describes the healthiest patient. The GAF is broadly acknowledged and has been applied worldwide in hundreds of studies (Aas 2010).
- To assess therapist variables, we will apply items of the Development of Psychotherapists Common Core Questionnaire (DPCCQ; (Orlinsky and Ronnestad 2005)). The full DPCCQ consists of 370 questions, and its psychometric properties have been demonstrated in a sample of about 5000 therapists (Orlinsky and Ronnestad 2005). We will apply the subscales of the instrument that have been found to be predictive of therapeutic processes and outcomes in several studies: difficulties in practice, warm interpersonal style, advanced relational skills, personal satisfaction, and personal burdens (Nissen-Lie et al. 2014).
- To assess general change mechanisms, we will apply the Scale for the Multiperspective Assessment of General Change Mechanisms in Psychotherapy (SACiP; (Mander et al. 2013)). The SACiP is a measure with six dimensions: resource activation; problem actuation; mastery; clarification of meaning; emotional bond, with three items each; and agreement on collaboration, which comprises the aspects of tasks and goals, with six items. The measure demonstrated an excellent factor structure (with 0.51 ≤ λ ≤ 0.85), revealed good internal consistencies (with 0.71 ≤ α ≤ 0.90), and was outcome predictive.
- To assess patients’ and therapists’ general development of mindfulness over the course of the study, we will apply the Kentucky Inventory of Mindfulness Skills (KIMS). The KIMS (Baer et al. 2004) is based on the conception of mindfulness described in dialectical behavior therapy (Linehan 1993a; Linehan 1993b) and addresses four aspects: observing, describing, acting with awareness, and accepting without judgment, with 39 items that are rated on a five-step scale. The EFA revealed an excellent factor structure with factor loadings of 0.41 ≤ λ ≤ 0.86; excellent internal consistencies, with 0.83 ≤ α ≤ 0.91; and is outcome predictive (Baer et al. 2004). Several studies replicated the four-subscale structure of the KIMS by means of factor analyses (e.g. (Ströhle et al. 2010; Baum et al. 2010)).

### Demographics and other process measures

- Standard measures of the demographic data of patients (education level, medication, duration of illness, partnership, former psychotherapy) and the Structured Clinical Interview for the DSM (SCID) according to the DSM-5 criteria (Falkai et al. 2014) will be applied. Additionally, we will assess the intensity of the patients and therapists mindfulness and PMR experiences prior to the study; as well as the current intensity of patients’ and therapists’ mindfulness and PMR exercises conducted outside of the therapy sessions. The intensity of the mindfulness and PMR experiences will be used as a control variable in subsequent data analyses. Furthermore, we will assess adherence to the interventions.
- To assess patients’ and therapists’ in-session therapeutic presence, we will apply the Therapist Presence Inventory (TPI; (Geller et al. 2010)). The instrument measures the in-session presence operationalized as being fully in the present moment with an attitude of acceptance and openness. An exploratory factor analysis (EFA) revealed an excellent one-factor structure. Furthermore, the measure revealed a good internal consistency (with α = 0.75) and predicted the outcome as well as the therapeutic alliance.
- As for adherence control concerning the brief interventions, we will apply the Practice Quality-Mindfulness (PQ-M; (del Re et al. 2013)). The six items of the PQ-M assess the perceived quality of mindfulness implementation that is operationalized as perseverance in (a) receptive and (b) present-moment attention. The measure showed good psychometric properties, with 0.72 ≤ α ≤ 0.87, and is outcome predictive.
- To test potential allegiance effects, we will apply an adapted version of the allegiance scale developed by Falkenström (Falkenstrom et al. 2013). The scale consists of 30 items that assess the personal and professional attitude of therapists and patients towards TAU-M, TAU-PMR, and TAU.
- To assess the application of specific therapeutic techniques, we will apply an instrument that has been implemented in the CPP during the last years that measures the intensity of the CBT techniques during the last weeks. The validity of the measure has been demonstrated (Löffler et al. in press).

### Study from the participant’s point of view

**Fig. 2:**
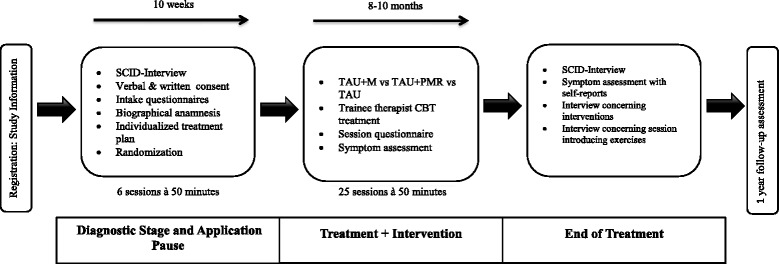
Study design from the participant’s point of view

The study design from the participant’s point of view is depicted in Fig. 2. The standard procedure at the CPP is that patients are listed on a waiting list after a diagnostic screening phone call. Then, they will be contacted by the study team and receive verbal and written information on the study.

Twice a year, a new group of trainee therapists at the CPP starts their outpatient therapies. Consequently, trainees receive verbal and written information on the study there. Trainees who participate in the study will then participate in the two mindfulness workshops described above.

The information given to the participants of the study includes a precise description of the inclusion and exclusion criteria, information concerning the interventions, questionnaires, and the data-collection procedure. Furthermore, it will be emphasized that study participation is on a voluntary basis and that there is the option to revoke consent to participate in the study at any time without having to cite reasons or suffer disadvantages. Moreover, participants have the opportunity to instruct the study team to delete their data without providing reasons.

### Statistical analysis

We will compare baseline descriptive statistics between the three study arms with *χ*^2^-tests (for categorical variables) and with ANOVA / t-tests (for continuous variables).

In line with the recommendations of Baldwin, Imel, Braithwaite, and Atkins (Baldwin et al. 2014), we will apply a multilevel modeling approach to address the nested data structure (sessions at level 1 are nested within patients at level 2, which are nested within therapists at level 3): Thereby, we will treat time as a within-subject factor and treatment condition as a between-subject factor. We will analyze main effects, that is, differences in intercepts of TAU + M versus TAU + PMR versus TAU concerning process and outcome variables, and interactive effects, that is, differences in slopes of TAU + M versus TAU + PMR versus TAU concerning process and outcome variables (Raudenbush and Bryk 2002). Our statistical hypothesis implies that the intercept is significantly higher in the TAU + M than in the TAU + PMR and the TAU concerning both the process and outcome variables. Additionally, it implies that the slope increases significantly stronger in the TAU + M than in the TAU + PMR and the TAU concerning both the process and outcome variables. We will conduct analyses on the intention-to-treat sample as well as on the completer sample. Furthermore, patients’ and therapists’ pre-treatment characteristics will be investigated as outcome predictors at levels 2 and 3 to control for differential effects on outcome in the three treatment arms.

## Discussion

Mindfulness, a specific form of attention that is non-judgmental, purposeful, and focused on the present moment, has its origins in an Eastern Buddhist tradition that is over 2500 years old (Cigolla and Brown 2011) and is currently well established in CBT (Kabat-Zinn 1990; Grossman et al. 2004). Usually, mindfulness interventions have been investigated in a structured, manualized group setting (Bohlmeijer et al. 2010). However, an increasing demand to investigate the effects of SIIME practiced together by patients and trainee therapists in individual therapy under effectiveness conditions has been highlighted, because this could elucidate opportunities to improve clinical training (e.g. Ryan et al. 2012; Bruce et al. 2010; Dunn et al. 2013; Grepmair et al. 2007). Furthermore, only a few studies have compared mindfulness interventions with an active control group, and most of the investigations that did were conducted with non-clinical samples. Most of these studies applied PMR as an active control group and demonstrated beneficial effects of mindfulness (e.g. Feldman et al. 2010; Semple 2010). Consequently, we designed the PrOMET study to analyze the effects of brief SIIME conducted by both trainee therapists and their patients at the beginning of individual therapy sessions in a randomized, controlled, longitudinal design under effectiveness conditions with TAU + PMR and TAU as control groups.

### Innovative aspects of the PrOMET study

With the PrOMET study, we intend to address four innovative aspects of effectiveness research: First, concerning the therapy session, our study is one of the first to investigate effects of different rituals of session introduction (TAU + M versus TAU + PMR versus TAU) in individual psychotherapy. Second, concerning the training of psychotherapists, we want to create direct intersections between workshop theory and practical clinical training by directly implementing mindfulness and PMR elements from the workshop training into individual therapy sessions of trainees and then investigate them in our research design. Third, concerning research strategies, we intend to combine in our naturalistic study elements from process-outcome research with aspects from the research designs of randomized, controlled trials. Fourth, concerning mindfulness, we will transfer elements of mindfulness that are traditionally investigated in group-therapy settings to individual therapy and investigate them scientifically.

### Aspects concerning bias minimization

In our nested study design, we will investigate patient and therapist contributions concerning the effects of the interventions. In contrast to double-blind medical trials, patients and therapists are informed about the different intervention groups and will actively participate in the study plan because they perform the interventions actively by themselves. This active integration of participants into the treatment plan should not be regarded as bias but rather as an important aspect of the successful implementation of psychotherapeutic interventions and as a necessary condition to implement the study design (Flückiger 2014). We will investigate the intensity of the active involvement of participants by asking specific questions concerning mindfulness and PMR experiences during the session and mindfulness and PMR practice at home and then statistically control these aspects as manipulation checks. Additionally, we will apply an allegiance scale that addresses positive and negative attitudes towards all three treatment conditions. The allegiance scale will be completed by patients and therapists and then used to control for potential bias effects of specific attitudes.

#### Bias minimization, patient

An experienced research psychotherapist who is not involved in the study will randomly assign patients to the three different treatment conditions to reduce the potential biases of participant characteristics. All researchers and participants of the study are blind to the randomization process. Furthermore, the specific inclusion and exclusion criteria define relatively homogenous groups. Additionally, comorbidities with other psychiatric disorders, age, sex, prior experiences with mindfulness and PMR techniques, and the intensity of the current mindfulness and PMR practice will be tested as potential confounding variables. A potential limitation is that it is not possible to blind the participants concerning the different treatment groups because they will know which treatment condition they are randomized to according to the specific intervention they receive during the therapy sessions. However, this is a typical limitation of most psychotherapy studies with a randomization process (Flückiger 2014). We addressed this issue, as described above, according to the revised CONSORT statement (Zwarenstein et al. 2008; Altman et al. 2001).

#### Bias minimization, therapist

The allegiances of therapists to the mindfulness or PMR approaches could be a potential bias (Munder et al. 2012). We will try to minimize this bias by randomizing patients (and not therapists) to the different treatment conditions in a crossed-therapist design (Falkenstrom et al. 2013). Consequently, each therapist potentially treats patients under all treatment conditions, since it is likely that different patients treated by the same therapist will be randomized to different treatment conditions. Additionally, we will apply an allegiance scale as described above. Therefore, allegiance biases will be statistically controlled. However, our sample size does not allow a perfect control for this effect. Consequently, it has to be noted as a potential limitation of the generalizability of the study results. Additionally, prior experiences with mindfulness and PMR techniques, as well as the intensity of the current mindfulness and PMR experience and practice with patients in therapy sessions, will be tested as potential confounding variables.

## Conclusion

In our study, we will investigate the effects of SIIME conducted by trainee therapists and their patients at the beginning of individual therapy sessions in a randomized, controlled, longitudinal design under effectiveness conditions with TAU + PMR and TAU as control groups. The study results could have important practical implications because they could inform ideas about how to improve the training of psychotherapists in outpatient training centers that could be implemented very easily, especially since there is no need for complex infrastructures or additional time concerning these brief SIIME that are directly implemented in the treatment sessions.

## Appendix A: Session-introducing intervention with mindfulness elements

You are sitting in the chair in a position that feels comfortable to you. You sit upright; your feet are flat on the floor. Your hands are resting on your upper legs. If it makes you feel good, you can allow your eyes to close gently [5-s break].

Throughout the exercise, please take an open-minded non-judgmental stance vis-à-vis everything you experience in the here and now [5-s break]. Focus all of your attention on your breath. Observe as the air is inhaled and exhaled [15-s break].

Now come into awareness with the physical sensations of your breath. Try to simply accept all of the experiences as they occur to you without judging them [15-s break]. By paying attention to your breath you will gradually advance into the here and now. You may now notice how your abdomen is lifted up as you inhale and how it is lowered again as you exhale. [15-s break].

Keep focusing your attention on your breath and always return to it even when your thoughts begin to wander. Try to encounter everything you experience with a friendly, accepting attitude [40-s break].

Now, expand your sphere of attention and simply become aware of the feelings in the rest of your body without making any attempt to alter them [15-s break]. Maybe you will become aware of your physical stance or the expression of your face [15-s break]. As you are doing this, open up to all of the experiences that unfold in the present moment. [40-s break].

Next, whenever you are ready, let go of these feelings. Slowly open your eyes with the intention of bringing this attention into the present moment and the rest of the day.

Duration: 4:47

## Appendix B: Control condition: Session-introducing progressive muscle relaxation exercise

You are sitting in the chair in a position that feels comfortable to you. You sit upright; your feet are flat on the floor. Your hands are resting on your upper legs. If it makes you feel good, you can allow your eyes to close gently [5-s break].

First, focus your attention on the muscles in both of your arms. Tighten both of your arms simultaneously – now – [5-s break] – and when you exhale the next time, let go and relax your arms. Focus intensively on the gradual release of tension and the sense of relaxation that ensues [20-s break].

Now, please focus your attention on the muscles in your face, neck and nape of the neck. Tighten up your face, neck and nape of the neck – all at the same time – now - [5-s break]. Then, as you exhale again, release the tension and relax. Every time you exhale, allow yourself to relax more deeply and even deeper the next time [20-s break].

Next, turn your attention on the muscles in your torso. Tighten up your shoulders, back and belly at the same time – now – [5-s break]. As you exhale again, allow the tension to be released and relax. Allow this release to flow down from the shoulder to the spine and down your entire back every time you inhale or exhale [20-s break].

At this point, focus your attention on your legs, please. Tighten up both legs simultaneously - now – [5-s break]. When you exhale again, let go of the tension and relax. Notice the pleasant feeling of relaxation, which gradually spreads through both legs [20-s break].

In the final exercise, please tension up all muscles in your entire body – now – [5-s break]. When you exhale again, let all of the tension go and relax. As you breath in and out several times, surrender yourself completely to the sense of relaxation and allow it flow through your whole body [20-s break].

Next, whenever you are ready, let go of these feelings. Slowly open your eyes with the intention of bringing this relaxation into the present moment and the rest of the day.

Duration: 4:47
